# Cytotoxicity effect of degraded and undegraded *kappa* and *iota* carrageenan in human intestine and liver cell lines

**DOI:** 10.1186/1472-6882-14-508

**Published:** 2014-12-17

**Authors:** Shahrul Hisham Zainal Ariffin, Wong Woan Yeen, Intan Zarina Zainol Abidin, Rohaya Megat Abdul Wahab, Zaidah Zainal Ariffin, Sahidan Senafi

**Affiliations:** Faculty of Science and Technology, School of Biosciences and Biotechnology, Universiti Kebangsaan Malaysia, 43600 Selangor, DE Malaysia; Department of Orthodontics, Faculty Dentistry, Universiti Kebangsaan Malaysia, 50300 Kuala Lumpur, Malaysia; Faculty of Applied Sciences, School of Biology, Universiti Teknologi MARA, 40450 Shah Alam, Selangor, Malaysia

**Keywords:** Cytotoxicity, Degraded, Undegraded, Carrageenan, Kappa, Iota, Apoptosis, Acid hydrolysis

## Abstract

**Background:**

Carrageenan is a linear sulphated polysaccharide extracted from red seaweed of the Rhodophyceae family. It has broad spectrum of applications in biomedical and biopharmaceutical field. In this study, we determined the cytotoxicity of degraded and undegraded carrageenan in human intestine (Caco-2; cancer and FHs 74 Int; normal) and liver (HepG2; cancer and Fa2N-4; normal) cell lines.

**Methods:**

Food grade *k*-carrageenan (FGKC), dried sheet *k*-carrageenan (DKC), commercial grade *k*-carrageenan (CGKC), food grade *i-*carrageenan (FGIC) and commercial grade *i*-carrageenan (CGIC) were dissolved in hydrochloric acid and water to prepare degraded and undegraded carrageenan, respectively. Carrageenan at the concentration range of 62.5 – 2000.0 μg mL^−1^ was used in the study. MTT assay was used to determine the cell viability while the mode of cell death was determined by May-Grunwald Giemsa (MGG) staining, acridine orange-ethidium bromide (AO/EtBr) staining, agarose gel electrophoresis and gene expression analysis.

**Results:**

Degraded FGKC, DKC and CGKC showed IC_50_ in 24, 48 and 72 hours treated Caco-2, FHs 74 Int, HepG2 and Fa2N-4 cell lines as tested by MTT assay. Degraded FGIC and CGIC only showed its toxicity in Fa2N-4 cells. The characteristics of apoptosis were demonstrated in degraded *k*-carrageenan treated Caco-2, FHs 74 Int, HepG2 and Fa2N-4 cells after MGG staining. When Caco-2 and HepG2 cells were undergone AO/EtBr staining, chromatin condensation and nuclear fragmentation were clearly seen under the microscope. However, DNA ladder was only found in HepG2 cells after gel electrophoresis analysis. Degraded *k*-carrageenan also inactivated PCNA, Ki-67 and survivin gene in HepG2. On the other hand, undegraded FGKC, DKC, CGKC, FGIC and CGIC treated cells showed no cytotoxic effect after analyzed by the same analyses as in degraded carrageenan.

**Conclusion:**

Degraded *k*-carrageenan inhibited cell proliferation in Caco-2, FHs 74 Int, HepG2 and Fa2N-4 cell lines and the anti-proliferative effect was related to apoptosis together with inactivation of cell proliferating genes as determined by morphological observation and molecular analysis. However, no cytotoxic effect was found in undegraded carrageenan towards normal and cancer intestine and liver cell lines.

## Background

Carrageenan, a naturally occurring anionic sulphated linear polysaccharide is extracted from red seaweed of the family Rhodophyceae. It has been used as a safe food additive for decades and can be found in soy milk, ice cream, yogurt, meats and even in toothpaste. In the review of 45 studies, Joanne Tobacman pointed out that the exposure to degraded or/and undegraded carrageenan causes intestinal lesions in different animal models [1]. Nevertheless, based on the short and long-terms studies of toxicology in rats, hamsters, guinea pigs and monkeys reviewed by the Joint FAO/WHO Expert Committee on Food Additives (JECFA), they concluded that the carrageenan is safe in diet and recommended an Acceptable Daily Intake of “not specified” for carrageenan [2]. As a result, carrageenan is continually used to improve texture and as gelling, stabilising and thickening agents [3].

The toxicology of carrageenan is related to the unique chemical structure of carrageenan. It has backbone formed by alternate units of D-galactose and 3,6-anhydro-galactose, joined by α-1,3 and β-1,4-glycosidic linkage [4]. From a commercial point of view, the most important carrageenans can be categorised into kappa (*k-*), iota (*i*-) and lambda (*λ*-) carrageenans, which differ in the number and position of the sulphate groups. *k-*carrageenan is a source from *Kappaphycus alvarezi* (cottonii) which has an ester sulphate content of about 25-30% and a 3,6-AG content of about 28-35%. *i-*carrageenan is produced from *Eucheuma denticulatum* (spinosum) which has an ester sulphate content of about 28-30% and a 3,6-AG content of about 25-30%. *k*-carrageenan form a stronger and rigid gels than *i*-carrageenan because lower level of ester sulphate has higher solubility temperature and stronger gel strength [5]. In food industry, normally 0.05 - 1% (w/v) of the *k-*carrageenan or *i*-carrageenan will be used in food [6, 7]. Carrageenan can also be categorized as high molecular weight, or “undegraded” carrageenan when dissolving in water. The low molecular weight carrageenan, or “degraded” carrageenan with average molecular weight of 10–20 kDa has been associated with inflammatory effect and induce tumours in experimental animals [4]. As a result, the FDA proposed regulations that commercial-grade carrageenan could not have a molecular weight under 100 kDa.

Sulphated polysaccharides from seaweed are known to have a variety of biological activities, especially the low molecular weight oligosaccharides [8–10]. The biological activities of sulphated polysaccharides include antiviral [11, 12], anti-oxidant [13], anticoagulant [14] and antitumour activities; the antitumour activities of polysaccharides may be due to immunotherapeutic properties that inhibit the growth of tumour cells [15]. One study revealed that the biological actions of sulphated polysaccharides are influenced by the number of sulphate groups and the molecular weight of the polysaccharide [16]. Meanwhile, degraded carrageenan has long been known to cause ulcerative colitis in rats and guinea pigs [17]. The degraded carrageenan sulphated oligosaccharides can be produced by oxidative degradation [18], radiation [19], enzymatic hydrolysis [20] and mild acid hydrolysis [21]. Although prior reviews reported that the food grade carrageenan is safe to the consumers, the cytotoxicity of the food grade carrageenan which was prepared differently in acid and water toward liver and intestine cells are presently unclear.

To determine the cytotoxic effects of degraded and undegraded carrageenan on cancer and normal human intestinal as well as liver cells, we exposed Caco-2, FHs 74 Int, HepG2 and Fa2N-4 cells to degraded and undegraded food grade *k*-carrageenan and *i*-carrageenan (FGKC & FGIC), dried sheet *k*-carrageenan (DKC), commercial grade *k-*carrageenan and *i*-carrageenan (CGKC & CGIC). The cell viability, cell morphology and apoptosis or necrosis was being investigated.

## Methods

### Preparation of degraded and undegraded *kappa*and *iota*carrageenan

The food grade *k*-carrageenan and *i-*carrageenan powder (FGKC & FGIC) samples in our study were generously provided by Tacara Sdn. Bhd., Tawau, Malaysia (TA150). The dried, sheet *Kappaphycus alvarezii* carrageenan (DKC) was kindly donated by the School of Chemical Sciences and Food Technology, Faculty of Science and Technology, National University of Malaysia. Commercial grade *k*-carrageenan and *i*-carrageenan powder (CGKC & CGIC) was purchased from Sigma, USA, and they were used as comparative control. Briefly, DKC was blended into small pieces in a blender and ground into a powder with a particle size of 0.25 mm. FGKC and DKC are Processed *Eucheuma* Seaweed (PES) composed mainly of water-soluble molecular carrageenan (*k*-carrageenan) and insoluble algal cellulose [22]. The cellular DNA, proteins, fats, sugars and heavy metals are normally removed during the production of PES. Carrageenan powder (20 mg) was degraded with 0.1 M HCl at 60°C in a water bath placed on a hot plate for 4 hours with continuous stirring. The reaction was stopped by adding 0.1 M NaOH, and the solution was adjusted to pH 7.0-7.2 to obtain a final concentration of 10 mg mL^−1^. Undegraded carrageenan was prepared in accordance with the procedure of other studies [23] with some modifications. Briefly, carrageenan powder was dissolved in hot deionized distilled water (70°C) with continuous stirring for 1 hour. The solution was centrifuged at 300 g for 5 min. The supernatant was purified by sterile filtering through 0.45 μm and 0.22 μm membrane filters.

### Cell culture

The human epithelial colorectal adenorcarcinoma cell line, Caco-2 (ATCC HTB-37™) was cultured in DMEM (Gibco, USA) and non-essential amino acid (PAA); normal human small intestine cell line, FHs 74 Int (ATCC CCL-241™) was maintained in Hybri-care medium (ATCC 46-X™) and epidermal growth factor human (Sigma); human hepatocellular carcinoma cell line, HepG2 (ATCC HB-8065) was grown in RPMI 1640 medium (Gibco, USA) while immortalized human hepatocytes cell line, Fa2N-4 (ATCC PTA-5566) was cultured in MFE Supporting Medium with Component B (Xeno Tech); each of the cell lines was supplemented with 10% (v/v) foetal bovine serum (FBS) from Gibco, USA and 1% (v/v) 10,000 U/mL penicillin-streptomycin (Gibco, USA). Cells were incubated at 37°C in a humidified atmosphere with 5% CO_2_.

### Cytotoxixity assay using MTT

The antiproliferative and cytotoxic effects of degraded and undegraded FGKC, DKC, CGKC, FGIC and CGIC on Caco-2, FHs 74 Int, HepG2 and Fa2N-4 cells were assessed by MTT assay. Cells were seeded into 96-well microplates at a concentration of approximately 10,000 cells per well (200 μL per well). The cells were treated with degraded or undegraded carrageenan at concentrations of 62.5, 125.0, 500.0, 1000.0 and 2000.0 μg mL^−1^ for 24, 48 and 72 hours. Tamoxifen (TAM) was dissolved in 20% DMSO and used as a positive control in the study. In each well, 20 μL of TAM was added to 180 μL of cells to achieve a final TAM concentration of 0.625 – 20.000 μg mL^−1^, and the concentration of DMSO in each well was less than 1%. Approximately 10% of 5 mg mL^−1^ of MTT was added to each well after the indicated period of treatment, followed by 4 hours of additional incubation. Next, the medium was carefully withdrawn, and 100 μL of 100% DMSO was added to dissolve the insoluble formazan. The optical density was measured at 570 nm on a Model 680 Microplate Reader (BioRad, USA). All solvents and chemicals used for MTT analysis were purchased from Sigma, USA. The percentage of cell viability was calculated by comparing the absorbance of degraded *k*-carrageenan in treated cells to the absorbance in untreated cells.

### Morphological observation under an inverted microscope

Approximately 2×10^5^ cells were seeded in 2 mL of culture medium in 6-well plates (BD Labware, England) and incubated with 5% CO_2_ at 37°C for 24 h. The cells were then treated with degraded FGKC, DKC and CGKC at the appropriate concentrations to produce 50% cell viability at 72 hours as pre-determined by the MTT assay. For undegraded carrageenan, the highest concentration with longest treatment period (72 hours) was used to test on the cells. Meanwhile, TAM at the IC_50_ values of each cells (72 hours IC_50_) were also used as positive control. The morphology of the treated cells was observed under an inverted microscope at 200× magnification. The observed morphology was compared to the morphology of untreated cells.

### May-Grunwald Giemsa staining

Cell morphology was observed by staining with May-Grunwald Giemsa after 72 hours of treatment. Cells in plates were washed with 1 × PBS and stained with May-Grunwald (BDH Chemical, Ltd.) for 4 minutes. The cells were then rinsed with sterile water and flooded with freshly prepared Giemsa stain solution (BDH Chemical, Ltd.) for 15 minutes. The cells were rinsed three times with sterile water, and any morphological changes were observed by using an inverted microscope (Nikon, TMS) at 100× and 200× magnification.

### Acridine orange-ethidium bromide (AO/EtBr) staining

Caco-2 and HepG2 cells were seeded at a density of 5 × 10^5^ cells mL^−1^ in T-25 flasks and treated with degraded and undegraded FGKC, DKC and CGKC, or the positive control, TAM, for 72 hours. After the cells were harvested by trypsinisation, they were washed with PBS. A 25 μL sample of cells was stained with 10 μL of a mixture (1:1) of acridine orange and ethidium bromide solution. The concentration of AO/EtBr are 100 mg mL^−1^ in 1× PBS. After staining, the cells were viewed under a fluorescence microscope at 200× magnification (Leica DM 2500).

### Determination of apoptosis using DNA fragmentation

Caco-2 and HepG2 cells treated with degraded FGKC, DKC and CGKC were lysed with lysis buffer (10 mM Tris–HCl, 5 mM EDTA, 200 M NaCl, 0.2% SDS) and incubated at 60°C for 5 min. The RNA in each sample was digested with 10 μL of RNase at 37°C for 1 hour, and proteinase K (more than 3 U uL^−1^) was added before incubating at 50°C for an additional 1.5 h. NaCl (5 M) was then added, and the samples were incubated on ice for 5 min to precipitate the protein. The cells were centrifuged for 15 min at highest speed at 4°C, after which supernatant was collected, and an equal volume of isopropanol was added to precipitate the DNA. Then, each sample was centrifuged again at 10,000 *g* for 10 min at 4°C. Supernatants were discarded, and the pellets were washed with 70% cold ethanol. The DNA sample was left to dry at room temperature before adding 1× TE buffer. Electrophoresis was carried out in a 1.0% (w/v) agarose gel for 1.5 h at 70 V. The gel was examined under UV transillumination following ethidium bromide staining to determine the extent of apoptotic DNA fragmentation.

### Determination of cell death using gene expression analysis

Reverse transcription polymerase chain reaction (RT-PCR) was used to analyze the presence of transcribed mRNA for cell proliferation gene marker. Total cellular RNA was extracted from Caco-2 and HepG2 cell lines which had been treated with the IC_50_ degraded carrageenan and highest concentration of undegraded carrageenan (2 mg mL^−1^), at the longest time point (72 hours) using Trizol reagent (Invitrogen, USA) according to the manufacturer’s instruction**.** The extracted RNA was dissolved in DEPC-treated water. The RNA concentration and sample purity was determined using spectrophotometer (Bio Photometer plus, Eppendorf). Two steps RT-PCR were conducted where 1 μg of total RNA was used in the first strand cDNA synthesis using RevertAid First Strand cDNA Synthesis Kit (ThermoScientific, USA). Subsequent PCR reaction was done utilizing Mastercyler Gradient (Eppendorf, Germany). The expression of cell proliferation markers, e.g. PCNA, MKI67 and BIRC5 or known as survivin were analyzed with GAPDH gene acting as the endogenous control. The expected amplicon sizes were shown in Table 1. Second strand cDNA synthesis was conducted using 1 uL oligo-dT primed cDNAs, 0.125 units GoTaq, 1× GoTaq reaction buffer, 2 mM MgCl_2_, 0.2 mM dNTP and 1 μM of sense and antisense primers. PCR amplification were under the following conditions: initial denaturation at 95°C for 2 min, denaturation at 95°C for 1 min, primer annealing as stated in Table 1, extension at 72°C for 45 second, and final extension at 72°C for 5 min. After amplification, the PCR products were separated by electrophoresis on 1.7% (w/v) agarose gel in 1× TAE buffer. The separated DNA fragments were visualized by ethidium bromide staining and photographed using the Alpha Imaging System (Alpha Innotech, San Leandro, CA, USA) under UV transillumination.

**Table 1:** **Primer sequences used in RT-PCR analysis**

Gene	Primer	Sequences	Expected size (bp)	Annealing temp. (°C)/time (s)	# of cycles
GAPDH	Forward	5′ CCATGGAGAAGGCTGGG 3′	195	55°C/30s	30
	Reverse	5′ CAAAGTTGTCATGGATGACC 3′			
PCNA	Forward	5′ TCCCACGTCTCTTTGGTGC 3′	155	60°C/30s	30
	Reverse	5′ TCTTCGGCCCTTAGTGTAATGAT 3′			
MKI67	Forward	5′ GGAAAGTAGGTGTGAAAGAAGAGG 3′	458	50°C/60s	30
	Reverse	5′ GCCTTTATCCTCATCTCCTGGTAC 3′			
Survivin	Forward	5′ GGCATGGGTGCCCCGACGTT 3′	439	62°C/60s	30
	Reverse	5′ AGAGGCCTCAATCCATGGCA 3′			

References: GAPDH [24]; PCNA [25]; MKI67 [26]; Survivin [27].

### Statistical assay

Data were expressed as the mean ± standard error from three independent experiments. The significance of any differences (p < 0.05) between untreated and treated cells was analysed by using Student’s *t*-test. The data of MTT assays was also statistically analysed using two-way ANOVA to evaluate the effect of time, concentration and the interaction between concentration and time on cell viability.

## Results

### Cytotoxic activity of carrageenan on cells by MTT assay

**Figure 1:**
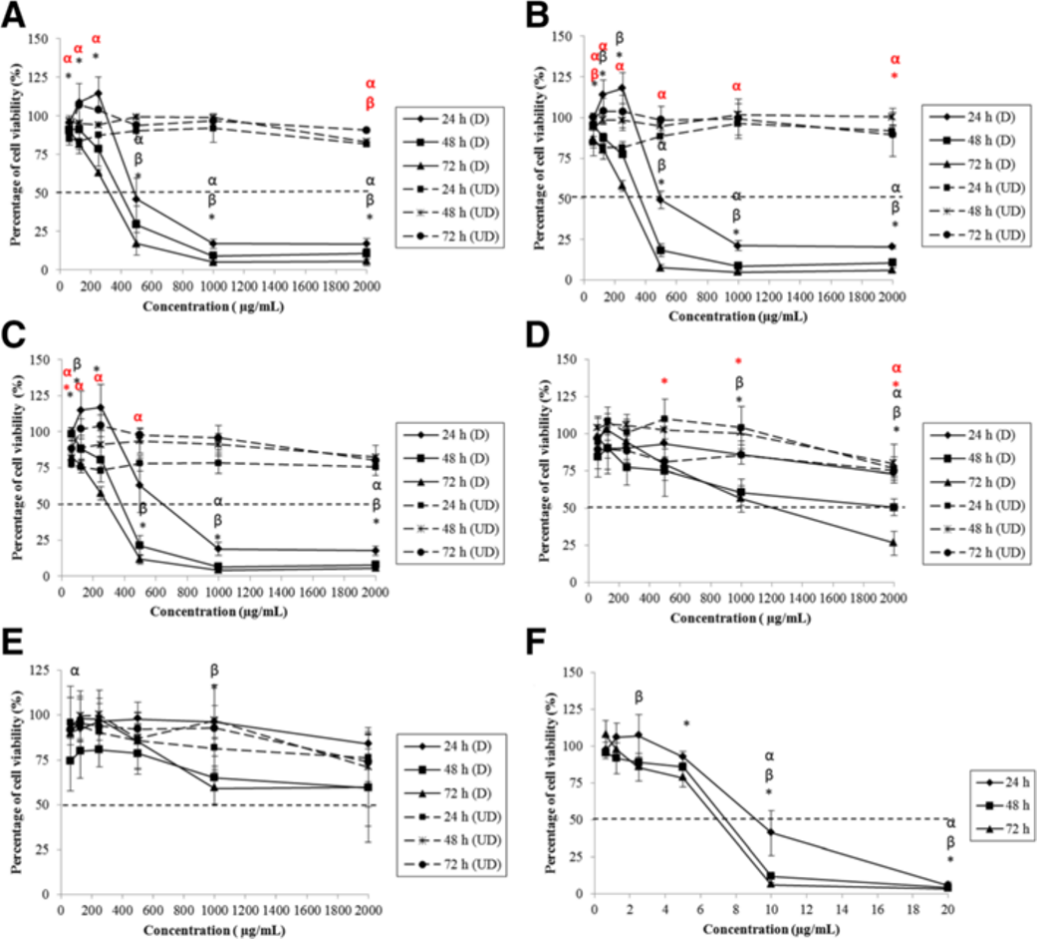
**Concentration and time effects of degraded and undegraded carrageenan in Caco-2 cell line, as measured by the MTT assay.** Caco-2 cells were treated with **(A)** FGKC, **(B)** DKC **(C)** CGKC **(D)** FGIC and **(E)** CGIC at concentrations of 62.5 – 2000.0 μg mL^−1^ and **(F)** tamoxifen at concentrations of 0.625- 20.000 μg mL^−1^ for 24 – 72 h. The percentages of cell viability were calculated by comparing the absorbance of treated and untreated cells. Degraded FGKC, DKC and CGKC showed IC_50_ in Caco-2 cells (black bold line) while IC_50_ values were not found in undegraded carrageenan treated cells (black dotted line). Each experiment had 3 replicates from 3 independent experiments (n = 3). The results are expressed as the mean ± SE. α: significant difference at 24 h; β: significant difference at 48 h and *: significant difference at 72 h for degraded carrageenan. Significant differences of undegraded carrageenan were in red symbols.

**Figure 2:**
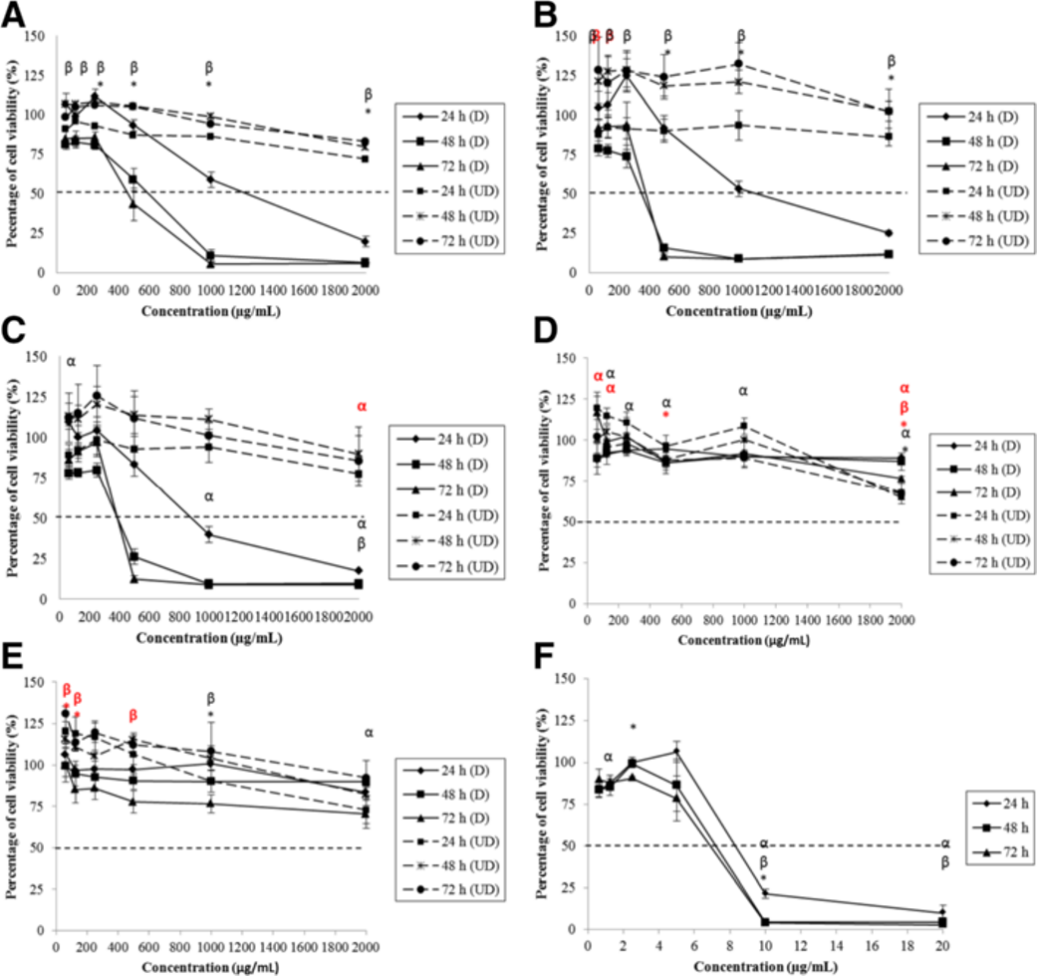
**Concentration and time effects of degraded and undegraded carrageenan in FHs 74 Int cell line, as measured by the MTT assay.** FHs 74 Int cells were treated with **(A)** FGKC, **(B)** DKC **(C)** CGKC **(D)** FGIC and **(E)** CGIC at concentrations of 62.5 – 2000.0 μg mL^−1^ and **(F)** tamoxifen at concentrations of 0.625- 20.000 μg mL^−1^ for 24 – 72 h. Degraded FGKC, DKC and CGKC showed IC_50_ in FHs 74 Int (black bold line). IC_50_ values were not found in degraded FGIC, CGIC and all undegraded carrageenan treated cells (black dotted line). Data are reported as means of three replicates from 3 independent experiments (n = 3). α: significant difference at 24 h; β: significant difference at 48 h and *: significant difference at 72 h for degraded carrageenan. Significant differences of undegraded carrageenan were in red symbols.

**Figure 3:**
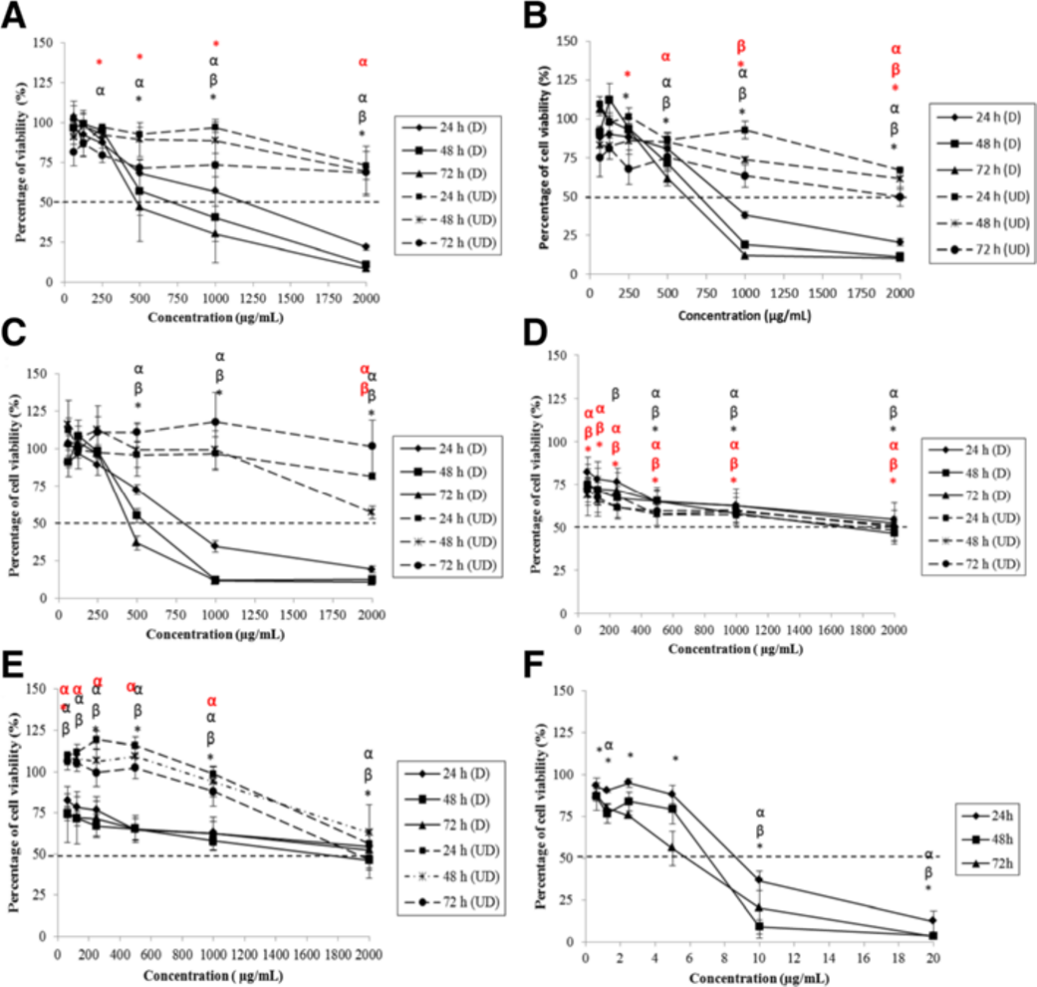
**Concentration and time effects of degraded and undegraded carrageenan in HepG2 cell line, as measured by the MTT assay.** HepG2 cells were subjected to the treatment of **(A)** FGKC, **(B)** DKC **(C)** CGKC **(D)** FGIC and **(E)** CGIC at concentrations of 62.5 – 2000.0 μg mL^−1^ and **(F)** tamoxifen at concentrations of 0.625- 20.000 μg mL^−1^ for 24 – 72 h. Degraded FGKC, DKC and CGKC also showed IC_50_ in HepG2 cells (black bold line). FGIC had IC_50_ > 1000 μg mL^−1^on the cells. Data are reported as means of three replicates from 3 independent experiments (n = 3). α: significant difference at 24 h; β: significant difference at 48 h and *: significant difference at 72 h for degraded carrageenan. Significant differences of undegraded carrageenan were in red symbols.

**Figure 4:**
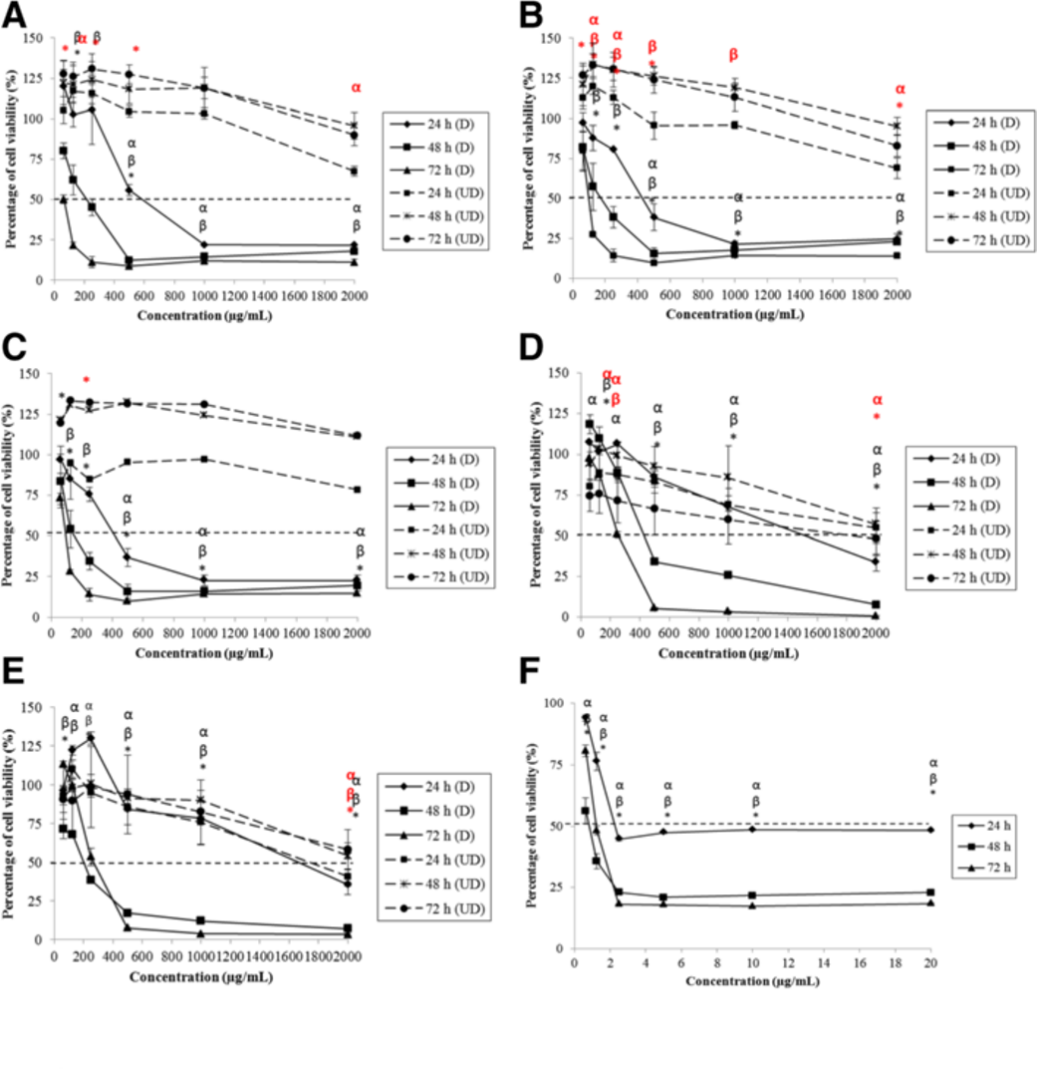
**Concentration and time effects of degraded and undegraded carrageenan in Fa2N-4 cell line, as measured by the MTT assay.** Fa2N4 cells were subjected to the treatment of **(A)** FGKC, **(B)** DKC **(C)** CGKC **(D)** FGIC and **(E)** CGIC at concentrations of 62.5 – 2000.0 μg mL^−1^ and **(F)** tamoxifen at concentrations of 0.625- 20.000 μg mL^−1^ for 24 – 72 h. All of the degraded carrageenan showing IC_50_ in Fa2N-4 cells (black bold line). Data are reported as means of three replicates from 3 independent experiments (n = 3). α: significant difference at 24 h; β: significant difference at 48 h and *: significant difference at 72 h for degraded carrageenan. Significant differences of undegraded carrageenan were in red symbols.

The cell viability of Caco-2, FHs 74 Int, HepG2 and Fa2N-4 treated by degraded and undegraded kappa (*k-*) and iota (*i-*) carrageenan was evaluated by 3-[4, 5-dimethylthiazol-2-yl]-2,5-diphenyl tetrazolium bromide (MTT). Figure 1A-C shows that Caco-2 cell viability decreased significantly (p < 0.05) in response to degraded FGKC, DKC and CGKC after 24, 48 and 72 hours of treatments. However, FGIC were not toxic to the cells with IC_50_ > 1000 μg mL^−1^ and no IC_50_ values of CGIC were found towards Caco-2 (Figure 1D and E). Two-way ANOVA analysis showed that the effect of time and concentration on cell viability of Caco-2 treated with degraded FGKC, DKC and CGKC was statistically significant (p < 0.05). The time and concentration interact significantly in the treatment of DKC and CGKC (p < 0.05). Compared to the negative control, the cells treated at pH 7 did not have any measurable IC_50_ values after 24, 48 or 72 hours. As positive control, tamoxifen (TAM) treatment yielded IC_50_ values of 9.2, 7.4 and 7 μg mL^−1^ after 24, 48 and 72 hours, respectively (Figure 1F). Degraded FGKC, DKC and CGKC, but not degraded FGIC and CGIC also inhibited the cell proliferation of FHs 74 Int significantly (Figure 2A-E). IC_50_ values of TAM were 8.4, 7.2 and 6.8 μg mL^−1^ (Figure 2F). The ANOVA was statistically significant, indicating that the cell viability was influenced by the treatment time and concentration (p < 0.05). Furthermore, a statistically significant interaction indicated that the effect of time on cell viability of FHs 74 Int depend on the concentration. IC_50_ values were also demonstrated in degraded FGKC, DKC and CGKC treated HepG2 cells. The percentages of cell viability decreased significantly (p < 0.05) in response to higher concentration (Figure 3A-C). However, different time points have no effect in HepG2 cells. Degraded FGIC and CGIC showed no cytotoxic effect in HepG2 cells with IC_50_ > 1000 μg mL^−1^ (Figure 3D and E). IC_50_ values of TAM were 9, 7 and 6 μg mL^−1^ (Figure 3F) whereas pH7 aqueous did not cause cell inhibition. Fa2N-4 cells with the treatment of degraded FGKC, DKC, CGKC, FGIC and CGIC yielded IC_50_ values after 24, 48 and 72 hours (Figure 4A-E). The effects of time and concentration were found dependent on each other with p < 0.05. IC_50_ values of TAM were 2.2, 0.8 and 1.2 μg mL^−1^ (Figure 4F). Table 2 summarises the IC_50_ values for 24, 48 and 72 hours treatments of Caco-2, FHs 74 Int, HepG2 and Fa2N-4 cells with degraded carrageenan. In liver cells, the IC_50_ values of the normal hepatocytes, Fa2N-4 were lower than the cancer hepatocytes, HepG2 in all treatments. However, IC_50_ values of cancer intestine cells, Caco-2 were low as compared to normal intestine cells, FHs 74 Int. None of the undegraded *k*-carrageenan (FGKC, DKC and CGKC) and *i*-carrageenan (FGIC and CGIC) showed cytotoxicity in cancer and normal intestine and liver cell lines with no IC_50_ values being observed in MTT assay (Figures 1, 2, 3 and 4).

**Table 2:** **Inhibition concentrations causing 50% cell death (IC**_**50**_ **) in the four cell lines**

Time sample	24 h				48 h				72 h			
	Caco-2	FHS 74 Int	HepG2	fa2N4	Caco-2	FHS 74 Int	HepG2	fa2N4	Caco-2	FHS 74 Int	HepG2	fa2N4
FGKC	480	1240	1150	580	44	600	700	200	320	460	500	63
DKC	500	1100	800	420	360	340	700	160	280	380	600	100
CGKC	640	880	800	420	380	380	550	160	280	380	450	100
FGIC	/	/	/	1500	1500	/	1400	440	1000	/	/	250
CGIC	/	/	/	1660	/	/	1640	200	/	/	/	260
TAM	9.2	8.4	9	2.2	7.4	7.2	7	0.8	7	6.8	6	1.4

IC_50_ values of degraded FGKC, DKC, CGKC, FGIC, CGIC and tamoxifen applied to cells for 24, 48 and 72 h. The negative control, cells treated with a pH7.0 solution, showed no cytotoxic effect toward the cells.

### Morphological observation under microscope

**Figure 5:**
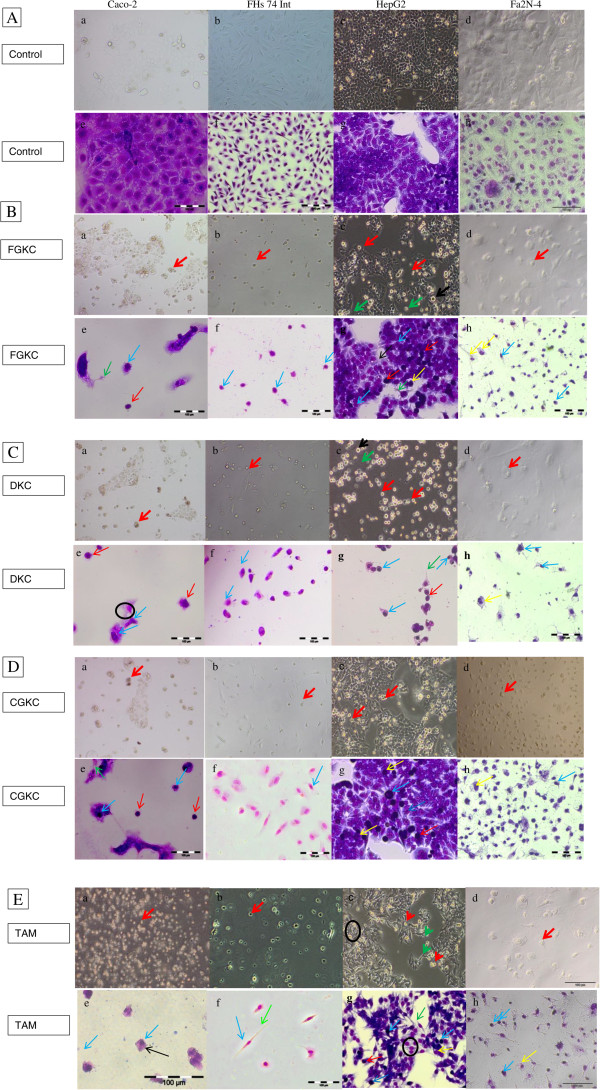
**Morphology observations before and after MGG staining of Caco-2, FHs 74 Int, HepG2 and Fa2N-4 cells following exposure to degraded** ***k*** **-carrageenan.** Cells were seeded at a cell density of 2 × 10^5^ cells per ml and incubated for 24 h. **A** (a-h) Untreated cells. **B** (a-h) Cells treated with FGKC; 5**C** (a-h) Cells treated with DKC; 5**D** (a-h) Cells treated with CGKC and 5**E** (a-h) Cells treated with tamoxifen at the IC_50_ values for 72 h were observed for apoptotic morphological characteristics. Treated cells showed chromatin condensation (blue arrow), microspike formation (green arrow), nuclear fragmentation (yellow arrow) and apoptotic bodies (red arrow).

The IC_50_ values of degraded FGKC, DKC, CGKC and TAM in different cell lines were used in the subsequent tests due to the 72 hours treatment contributed to a lower IC_50_ values. The morphologies of untreated and treated cells were observed under an inverted microscope before staining. The untreated Caco-2, FHs 74 Int, HepG2 and Fa2N-4 cells appeared to have normal epithelial morphology. The cells adhered to the surface of 6 well plates and the plasma membrane sticked tightly with the neighbouring cells as shown in Figure 5A (a), (b), (c) & (d). Cells treated with degraded FGKC, DKC or CGKC lost their surface attachment ability; they rounded up and floated in the medium. In addition, cell shrinkage was observed (Figure 5B (a-d), C (a-d), D (a-d) & E (a-d)). Some of the floating cells were found to be swollen. However, cells treated with undegraded FGKC, DKC and CGKC showed the same morphology as untreated cells. TAM at the IC_50_ values of each cell lines also caused cell shrinkage, and the cells lost their adherence and showed membrane blebbing as in Figure 5E (a-d).

### May-Grunwald Giemsa staining

Morphological examination can easily be performed by using May-Grunwald Giemsa staining to determine the mod of cell death [28–31]. Treated cells in 6-well plates were stained with the dye, and their morphologies were observed. The cytoplasm was stained blue while the nuclei were stained violet. The administration of degraded FGKC, DKC and CGKC to Caco-2 cells (320, 280 and 280 μg mL^−1^); FHs 74 Int (460, 380 and 380 μg mL^−1^); HepG2 cells (500, 600 and 450 μg mL^−1^) and Fa2N-4 cells (63, 100 and 100 μg mL^−1^) was found to induce cell death after 72 hours. These selected concentrations were according to the IC_50_ values obtained from MTT assay, which caused 50% of cell inhibition after the treatment. Degraded FGKC, DKC and CGKC treated cells demonstrated the characteristic of apoptosis such as membrane blebbing (black arrow), microspike (green arrow), chromatin condensation (blue arrow), nuclear fragmentation (yellow arrow) and apoptotic bodies (red arrow). Figure 5B-D (e), (f), (g) and (h) show Caco-2, FHs 74 Int, HepG2 and Fa2N-4 treated cells, respectively. In contrast, Figure 5A (e), (f), (g) and (h) show that the untreated Caco-2, FHs 74 Int, HepG2 and Fa2N-4 cells, which have normal morphology and still, attached to the well plates. Cells treated with undegraded FGKC, DKC and CGKC at the highest concentration (2000 μg mL^−1^) showed similar morphology with untreated cells and they were growing to about 70% confluence even after 72 hours (figures not shown). Any appearance of apoptotic morphologies was not being observed.

### Acridine orange and ethidium bromide (AO/EtBr) staining

**Figure 6:**
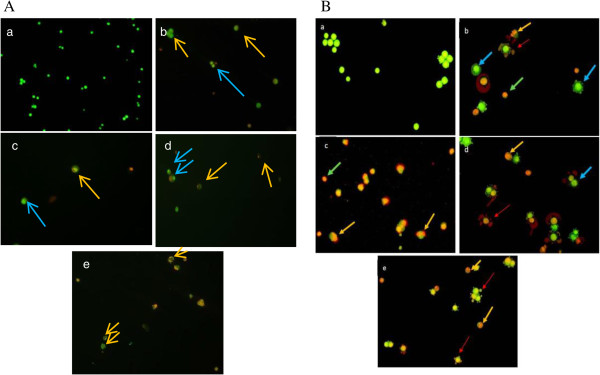
**Caco-2 and HepG2 treated cells stained with acridine orange and ethidium bromide.** Figure **A** shows Caco-2 cells while figure **B** shows HepG2 cells. (a) The untreated live cells showed normal green nuclei. (b) FGKC, (c) DKC, (d) CGKC and (e) tamoxifen-treated cells showed apoptosis, with bright green condensed nuclei and orange nuclei. Orange arrow: nucleus fragmentation. Blue arrow: chromatin condensation. Red arrow: apoptotic bodies. Light green arrow: necrotic cells.

Only Caco-2 and HepG2 cells were subjected to the following analyses because of the high proliferation rate of cancer cell lines as compared to normal cell lines. The morphologies of Caco-2 and HepG2 cells were shown in Figure 6A (b-d) and B (b-d), respectively. Following AO/EtBr staining, the nuclei of untreated Caco-2 and HepG2 cells had normal green colour when observed under the fluorescence microscope at 200x magnification as shown in Figure 6A (a) and B (a), respectively. After treated with degraded FGKC, DKC and CGKC, Caco-2 and HepG2 cells undergoing early apoptosis had bright green nuclei with condensed or fragmented chromatin (blue arrow); late apoptosis cells displayed condensed or fragmented orange chromatin (orange arrow) and apoptotic bodies (red arrow). Some of the HepG2 cells were found died by necrosis which demonstrated structurally normal orange nuclei (light green arrow). Tamoxifen (TAM), as a positive control, also caused apoptosis in Caco-2 with most of the cells showed nuclear fragmentation and in HepG2 cells which is identified by the characteristic of nuclear fragmentation and formation of apoptotic bodies.

### Gel electrophoresis analysis

**Figure 7:**
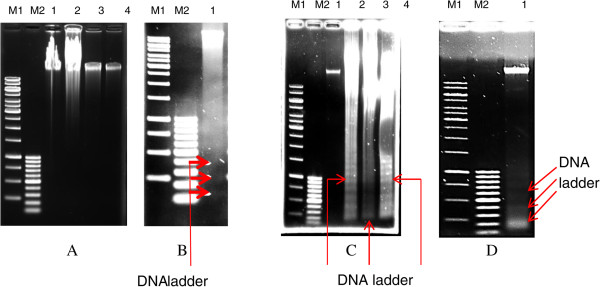
**Agarose gel electrophoresis of DNA extracted from untreated and treated HepG2 and Caco-2 cells.** Panel **A** and **B** demonstrate results from Caco-2 while Panel **C** and **D** show results from HepG2 cells. Caco-2 treated cells did not show any DNA fragments ladder. The genomic DNA of untreated Caco-2 and HepG2 cells was intact, whereas DNA fragmentation with a ladder pattern of ~180 bp was found in all degraded *k*-carrageenan HepG2 cells. DNA of Caco-2 and HepG2 cells fragmented after induced by tamoxifen (Panel **B** and **D**). Lane M1 shows a 1 kb DNA ladder, and lane M2 shows a 100 kb DNA ladder. Panel **A** &**C**; lane 1: untreated cells, lane 2: degraded FGKC-treated cells, lane 3: degraded DKC-treated cells, lane 4: degraded CGKC-treated cells. Panel **B** &**D**; lane 1: Tamoxifen-treated cells.

DNA fragmentation analysis was also performed to determine the amount of intrinsic apoptotic cell death. As shown in Figure 7A; lane 2, 3 & 4, DNA fragment ladder was not seen in degraded *k*-carrageenan treated Caco-2 cells. The untreated cells showed intact genome in Figure 7A; lane 1. However, DNA fragmentation was found to have occurred through intrinsic apoptosis in HepG2 cells when induced with degraded FGKC, DKC or CGKC. The cleavage of DNA produced DNA fragment ladders with band lengths representing integer multiples of nucleosome length (180–200 bp), as shown in Figure 7C; lanes 2, 3 and 4 on a 1% (w/v) agarose gel, whereas untreated cells showed intact genomes (Figure 7C; lane 1). Figure 7B and D show DNA fragmentation of Caco-2 and HepG2 cells treated with TAM as positive control. There was also no DNA fragmentation being observed in undegraded *k*-carrageenan treated Caco-2 and HepG2 cells.

### Gene expression analysis

**Figure 8:**
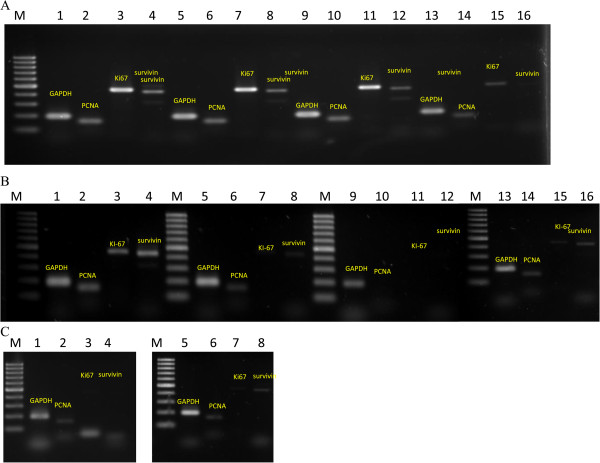
**Gene expression of proliferating cell nuclear antigen (PCNA), MKI67 and survivin analyzed by RT-PCR. (A)** Gene expression of Caco-2 cells treated with degraded FGKC, DKC and CGKC. **(B)** Gene expression of HepG2 cells treated with degraded FGKC, DKC and CGKC. **(C)** Gene expression of Caco-2 and HepG2 cells treated with tamoxifen. GAPDH was used as the control gene. Degraded *k*-carrageenan was found inactivate the proliferating gene in HepG2 cells but not in Caco-2 cells. **(A)** &**(B)** Lane 1–4: untreated cells. Lane 5–8: FGKC. Lane 9–12: DKC and lane 13–16: CGKC. Lane 1, 5, 9 & 13: GAPDH. Lane 2,6,10% 14: PCNA. Lane 3,7,11 & 15: MKI67. Lane 4, 8, 12 & 16: Survivin. **(C)** Lane 1–4: Caco-2 cells. Lane 5–8: HepG2 cell.

In order to determine the proliferative activity and apoptosis of degraded and undegraded *k*-carrageenan treated Caco-2 and HepG2 cells, RT-PCR was used to evaluate the gene expression of proliferating cell nuclear antigen (PCNA), MKI67 and survivin. Housekeeping gene GAPDH was used as endogenous control. Both cell lines treated with degraded *k*-carrageenan (FGKC, DKC and CGKC) at IC_50_ and undegraded *k*-carrageenan at the highest concentration (2000 μg mL^−1^) for 72 hours were subjected to RT-PCR analysis. Tamoxifen treated HepG2 and Caco-2 cells with IC_50_ value at 6 μg mL^−1^ and 7 μg mL^−1^, respectively was used as positive control for the treatment. PCNA, MKI67 and survivin are expressed in untreated, degraded FGKC, DKC and CGKC treated Caco-2 cells (Figure 8A). In HepG2 cells, the RT-PCR result showed that the gene expression of PCNA (Figure 8B; lane 6, 10, 14), MKI67 (Figure 8B; lane 7, 11, 15) and survivin (Figure 8B; lane 8, 12, 16) were inactivated by the treatments of degraded FGKC, DKC and CGKC, respectively. Lane 1, 2, 3 and 4 in Figure 8B show the gene expression of untreated cells. The lowest expression of the three genes was found in DKC treated HepG2 cells. On the other hand, undegraded *k*-carrageenan treated HepG2 and Caco-2 cells also expressed all the gene markers after treatment. PCNA, MKI67 and survivin gene expression in tamoxifen treated cells was lower than control Caco-2 and HepG2 cells (Figure 8C).

## Discussion

Carrageenan has been widely used in food, pharmaceutical and industrial applications. The commercial uses of carrageenan were reviewed by Weiner, 1991 [6]. Although carrageenan is classified as generally recognised as safe (GRAS) by the United States Food and Drug Administration, some researchers question the safety of ingested carrageenan, as it may be altered by bacteria in the intestine as long as the acidic conditions in the stomach [32, 33]. The present study was aimed to investigate the cytotoxic effect of degraded due to acidic exposure similar to stomach and undegraded for both *k*-carrageenan and *i*-carrageenan on normal and cancer human using intestine and liver cell lines *in vitro*.

Review from Tobacman demonstrated that the occurrence of intestinal ulcerations and neoplasms is related to the exposure to undegraded and degraded carrageenan [1]. In order to mimic *in vivo* cells, human intestinal Caco-2 and FHs 74 Int cell lines were used as a model of intestinal barrier [34, 35]. Besides, liver cells have vital role in metabolizing drug in mammals [36]. Liver samples from rats fed with 25% native carrageenans (kappa/lambda from *Chondrus crispus* or *iridaea*) in the diet for one month showed that the *C. iridaea* was stored in the liver in two animals, as determined by the presence of gamma metachromatic reaction sites in the Kupffer cells [37]. Gross observation of the livers tissue of rats receiving 5% kappa carrageenan from *I. cristapa* showed decrease size, rough surface and vascularization in 10/13 rats [38]. Therefore, the cellular level on cytotoxic effect of carrageenan in liver cells is vital to be investigated. The HepG2 cell line, which is a human hepatocarcinoma cell has been used in many cytotoxicity studies [39–41] because it can synthesize and secrete many of the plasma proteins characteristic of normal human liver cells [42]. However, the drug metabolizing enzymatic activity is lower than normal liver since HepG2 is a derivative from human hepatoblastoma [43, 44]. Fa2N-4 is normal liver cell line that retains the normal hepatocellular morphology and expression and inducibility of CYPs and transporter [45].

Food grade carrageenan and dried sheet *k*-carrageenan provided by Tacara is a semi-refined carrageenan where the cellulose was not removed during the extraction process. In contrast, commercial grade carrageenan supplied by Sigma is a pure carrageenan which involved alkali treatment, hot extraction, filtration, KCl precipitation or alcohol precipitation, dehydration, dried and then milled to get carrageenan powder [46]. Since carrageenan is water soluble polysaccharide, distilled water was used to dissolve the powder in order to produce undegraded carrageenan. Carrageenans can be depolymerised in acidic solutions, yielding low molecular weight (<20,000 g mol ^−1^) carrageenan, which can cause lesions. To simulate human gastric conditions, 0.1 M hydrochloric acid (pH1.0) was used to degrade carrageenan. Mild acid hydrolysis was used in the study to produce degraded carrageenan that results in highly specific cleavage [21]. Degraded carrageenan was prepared according to the acid hydrolysis method used in a previous study, with some modification [47]. Basically, the cytotoxic effects of compounds can be classified into a small number of categories according to their IC_50_ values. IC_50_ values less than 100 μg mL^−1^ indicate a potentially cytotoxic compound. IC_50_ values in the range of 100–1000 μg mL^−1^ are considered to correspond with moderate cytotoxic effects, and compounds with IC_50_ values greater than 1000 μg mL^−1^ are considered non-toxic to the cells [48–50]. In this study, degraded FGKC, DKC and CGKC were found to cause moderate toxic effects on Caco-2, FHs 74 Int, HepG2 and Fa2N-4 cells because the IC_50_ values of all cells were in the range of 100–1000 μg mL^−1^ (Table 2), except for the 24 hours treatment with FGKC and DKC on FHs 74 Int; and 24 hours treatment of FGKC on HepG2, which had IC_50_ greater than 1000 μg mL^−1^. At 48 and 72 hours, degraded FGIC and CGIC were found to cause IC_50_ values in Fa2N-4 cells only. Interestingly, all five types of undegraded *kappa* and *iota* carrageenan did not show any IC_50_ values on cancer and normal human intestine and liver cell lines. These results indicated that the molecular weight and degradation of polysaccharides have adverse effects on the cells. Our finding is supported by another study who found that the oligosaccharide fraction from *k*-carrageenan exhibits higher antitumour activity than polysaccharides against human nasopharyngeal carcinoma, gastric carcinoma and the HeLa cell line *in vitro* [47]. Our results also agree with the finding of Haijin M, Xiaolu J and Huashi G [51], in which the low-molecular-weight carrageenan of 1726 was effective in inhibiting Sarcoma 180 tumour cells *in vivo*. Non-sulfonated or light-sulfonated *kappa* carrageenan oligosaccharides were found to have higher antitumour activity than high-sulfonated carrageenan. Therefore, the study suggested that the biological activity of carrageenan oligosaccharides depends on the charge density related to the sulphate groups, the molecular weight and the spatial structure. Carrageenan is a large polysaccharides in which the undegraded form of carrageenan have molecular weight of 200–800 kDa [52], while the degraded carrageenan have low molecular weight of 10–20 kDa [4]. Undegraded carrageenan did not show cytotoxic effect on cancer and normal cells in our study. It seems possible that this result is due to the difficulty of the cells to absorb or transport large polymers into cells [51, 53]. On the other hand, the degraded form of carrageenan can form interaction with the cells thus showing cytotoxicity activity. Sialyl Lewis X and sialyl Lewis A oligosaccharides are known as “tumour associated antigens” because their expression levels were found to be increased in tumour cells [54, 55]. It has been suggested that carrageenan oligosaccharides may be able to recognise and interact with sialyl Lewis X or sialyl Lewis A on the tumour cell surface by way of carbohydrate-to-carbohydrate interactions [47]. As a consequence, carrageenan prevents the cancer cells from interacting with the basement membrane, which would inhibit cell proliferation and prevent adhesion to different substrates. As seen in Figure 6, cells detached from the plate which might be related to the degraded carrageenan that inhibit the adhesion between the cells and substrate. Tamoxifen is an anti-oestrogen that has been used as a first line treatment for breast cancer. Tamoxifen has also been used in therapies for other malignant cancers, such as hepatocellular carcinoma, which is oestrogen receptor-α-negative. Tamoxifen was used as the positive control in this study because it has been shown to induce apoptosis in HCCs [56–58] and Caco-2 [59]. As shown in Figures 1F, 2F, 3F and 4F, tamoxifen inhibited cell proliferation at low concentration in the range of 0–10 μg/mL in all the four cell lines. The tamoxifen-induced apoptosis pathway is thought to depend on the down-regulation of survivin expression as well as telomerase activity in HepG2 cells [57, 60]. In order to detect the mode of cell death of apoptosis, morphological features of apoptosis (such as compaction and fragmentation of cell nucleus) is a highly relevant method [61]. During early apoptosis, the cells round up, separate from neighbouring cells and then shrink after the cleavage of lamins and actin filaments in the cytoskeleton. Cell detachment is an early indicator of cell death through apoptosis, and it was observed in a study of the antiproliferative effects of ginseng saponins on human prostate cancer cells [62]. In this study, cell detachment was being observed following the treatment of degraded *k-*carrageenan (Figure 5B, C and D). Another study used May-Grunwald-Giemsa staining as well to determine the intrinsic anticarcinogenic effects of *Piper sarmentosum* ethanolic extract on a human hepatoma cell line, and the apoptosis morphologies were observed [63]. In another prior study to distinguish cell death induced by apoptotic and necrotic treatments, morphological criteria was used by observing the treated human carcinoma Hela cells under light (phase contrast, fluorescene) and scanning electron microscopy [64]. In our study, treatment with degraded FGKC, DKC and CGKC resulted in similar apoptosis morphologies. All of the morphological characteristics (membrane blebbing, chromatin condensation (pyknosis), nuclear fragmentation (karyorrhexis) and apoptotic bodies) except cell swelling are the hallmark of apoptosis [65]. The above characteristic can be seen in Figure 5B, C and D. In addition, the apoptotic cells formed microspikes (echinoid protrusions) [66] and showed DNA fragmentation in an internucleosomal pattern at ~200 base pairs (bp) in HepG2 [67]. Necrosis, in contrast, results in cellular swelling, organelle swelling, cytoplasmic blebbing or blister formation in addition to condensation of nuclear chromatin and an increase in membrane permeability as a result of depleting energy, which caused by the injury of the ionic pumps of the cell membrane [68]. Because the cell death of the HepG2 cells treated with degraded *k-*carrageenan is accompanied by cell shrinkage and cell swelling as shown in figure 5B (c) & C (c), apoptosis and necrosis most likely occurred after the exposure to degraded *k*-carrageenan. Further morphological and biochemical analyses were carried out to confirm the mode of cell death.

The results from our study suggested that the degraded *k*-carrageenan induced cell death which may relate to the apoptosis in Caco-2 and HepG2 cell lines were further confirmed by fluorescence staining. Acridine orange and ethidium bromide staining can be used to detect apoptosis [69]. Acridine orange can permeate the cells, thus causing the nucleus of cells to appear green. Treated cells lose their membrane integrity. As a result, ethidium bromide can enter the cells, and the nuclei are eventually stained orange as seen in Figure 6 [70]. The necrotic cells that were observed may be due to late-stage apoptosis, at which point the cells finally enter necrosis after a long period of treatment (72 hours). The findings of the current study are consistent with those of other studies that showed that oxidative degradation products of *k*-carrageenan (k-CODP) cause cellular toxicity in Caco-2 cells. The toxicity of k-CODP was suggested to act through the apoptosis pathway and the inflammatory response by causing the production of reactive oxygen species [18]. Nevertheless, another study indicated that carrageenan-induced cell death occurs through necrosis. The human colonic epithelial cell line NCM460 and primary human colonic epithelial cells were exposed to 1-10 mg/L of undegraded *λ*-, *k*- and *i*-carrageenan, and they showed increased cell death, reduced cell proliferation and cell cycle arrest. There was no DNA ladder pattern found in cells treated with carrageenan. However, poly (ADP-ribose) polymer Western blot analysis showed unchanged band densities for uncleaved PARP with an increase in the band density for 50 kDa fragments, providing evidence of necrosis [71].

When cells die through the intrinsic apoptosis pathway, the chromatin condenses and aggregates into dense compact masses in the nucleus. Endonucleases cleave the chromatin DNA into internucleosomal fragments of approximately 180 bp. The DNA fragmentation can subsequently be analysed by a DNA laddering assay. Some studies found that DNA fragmentation happened at a later time during apoptosis, after the formation of separated apoptotic bodies or after final cell lysis [65, 72]. Therefore, the absent of DNA fragment ladder observed in Caco-2 cells following degraded *k*-carrageenan treatment (Figure 7A) may due to the Caco-2 cells were in the early stage of apoptosis, or the concentration of the treatment were not enough to induce DNA fragmentation. HepG2 cells undergone late phase of apoptosis with DNA ladder pattern of 180 bp as shown in Figure 7C, and with a small proportion of necrotic cells as determined by morphological and biochemical analysis. DNA fragmentation with a ladder pattern as the hallmark of apoptosis was demonstrated in the other studies as well [73–75]. TAM as a positive control was also proven to induce apoptosis in estrogen- receptor-negative cell lines by displaying DNA ladder pattern [59, 63, 56]. This molecular analysis further supported the results of cell viability and morphological observation of HepG2 treated cells.

In gene expression analysis by RT-PCR, the same concentration of RNA, i.e. 1 μg from treated and untreated cells were used to synthesis first and second strand cDNA. Meanwhile, same cycle number (30 cycles) was applied during the PCR amplification steps. Following gel electrophoresis, the expression of mRNA from treated cells was compared with the control cells. GAPDH was used as the internal control to normalize for samples to sample variations in total RNA amounts. Wang X, Xia Y, Liu L, Liu M, Gu N, Guang H and Zhang F [3] suggested that RT-PCR analyses can supplement the MTT assay results in evaluating the cytotoxicity of prosthodontic materials. In our study, the expression of PCNA in all undegraded *k*-carrageenan treated Caco-2 and HepG2 cells, as well as Caco-2 treated with degraded *k*-carrageenan, indicated that the cells passed the first checkpoint, which is G_1_ phase and eventually entered S phase followed by G_2_/M phase. However, degraded *k*-carrageenan inhibited the proliferation of HepG2 cells as PCNA mRNA was shown inactivated as compared to negative control (Figure 8B). PCNA is a nuclear antigen whose expression is restricted to proliferating cells. It is a well-known cell proliferation marker. It plays an important role in the process of DNA replication. PCNA expression will start to increase in late G_1_ phase with the highest expression in S-phase and at sites of ongoing DNA replication, and gradually decreases during G_2_ and mitosis of cycling cells. In quiescent cells, PCNA expression was low to undetectable [76]. Research from Zhu C, Cao R, Zhang S-X, Man Y-N and Wu X-Z [77] showed that fucoidan, a sulphated polysaccharide purified from brown algae significantly inhibited the tumour growth and the expression of PCNA. So, the inhibition of cell growth may due to the low expression of PCNA. Our study demonstrated that MKI67 gene is expressed in undegraded FGKC, DKC and CGKC treated Caco-2 and HepG2 cells, but undetectably low in tamoxifen and degraded *k*-carrageenan HepG2 treated cells. When the proliferation of HepG2 cells were restricted and undergone apoptosis following treatment such as *N*-benzoyl-12-nitrodehydroabietylamine-7-one (compound **81**), expression level of MKI67 was suppressed as determined by Lin L-Y, Bao Y-L, Chen Y, Sun L-G, Yang X-G, Liu B, Lin Z-X, Zhang Y-W, Yu C-L and Wu Y [78]. MKI67 is a nuclear antigen involved in the maintenance of cell proliferation and is expressed in G_1_, S, G_2_ and mitotic phases of cell cycles [26]. According to the RT-PCR result, the undegraded *k*-carrageenan treated Caco-2 and HepG2 cells were able to undergo all cell cycle events while in tamoxifen and degraded *k*-carrageenan treated cells, most of the cells stopped proliferating. This means that MKI67 also play a role in response to degraded *k*-carrageenan treatment. Cytotoxic effect of *k-*carrrageenan was further confirmed by the level of gene expression of survivin. Survivin function in inhibiting cells from apoptosis as well as regulating cell division [25]. The survivin gene is expressed in all undegraded FGKC, DKC and CGKC treated Caco-2 and HepG2 cells. Survivin is not expressed in degraded *k*-carrageenan treated HepG2 cells, Caco-2 and HepG2 cells which have exposed to tamoxifen at 7 and 6 μg mL^−1^, respectively. This finding was coincided with the MTT and May Grunwald Giemsa staining results, showing that degraded *k*-carrageenan and tamoxifen induced HepG2 cell death through apoptosis pathway by down-regulating survivin expression [60].

Studies of the *in vivo* antitumour and immunomodulation activities of *k*-carrageenan and *λ*-carrageenan suggest that the antitumour effects are promoted by the immune system. For example, carrageenan oligosaccharides enhance humoral and cell-mediated immunity [15, 79]. Additionally, the antitumour effect may be related to the antioxidant activity of sulphated polysaccharides [13]. Degraded *k*-carrageenan could be a useful compound for inhibiting tumour cell growth. This is because the degraded form of *k*-carrageenan can trigger cell death through apoptosis, as determined by morphological and molecular level analysis.

## Conclusion

In conclusion, the mild acid hydrolysis products of *k*-carrageenan showed moderate cytotoxic effects to both intestine (Caco-2; cancer and FHs 74 Int; normal) and liver (HepG2; cancer and Fa2N-4; normal) cell lines. The mode of cell death is suggested to be through intrinsic apoptosis HepG2 cells, as determined by morphological observations and molecular analysis. Meanwhile, undegraded carrageenan was found not toxic to the four cell lines as determined *in vitro.*

## Abbreviations

bp: base pair
CGKC: Commercial grade kappa carrageenan
FGKC: Food grade kappa carrageenan
g: gravity
h: hours
HCC: Hepatocellular carcinoma cell
*i-*: iota
*k-*: kappa
mL: milliliter
DKC: Dried kappa carrageenan
U: unit
μg: microgram.
